# Mitochondrial Dysfunction in Attention Deficit Hyperactivity Disorder

**DOI:** 10.5152/eurasianjmed.2022.22187

**Published:** 2022-12-01

**Authors:** Hakan Öğütlü, Meryem Kaşak, Selin Tutku Tabur

**Affiliations:** 1 Department of Child and Adolescent Psychiatry, Cognitive Behavioral Psychotherapies Association, Ankara, Turkey; 2 Department of Child and Adolescent Psychiatry, Ankara City Hospital, Ankara, Turkey; 3 Department of Psychology, Hasan Kalyoncu University Faculty of Economics, Administrative and Social Sciences, Turkey

**Keywords:** Attention deficit hyperactivity disorder, ADHD, mitochondrial dysfunction, mitochondrial DNA, mtDNA

## Abstract

Attention deficit hyperactivity disorder is a neurodevelopmental disorder with primary symptoms of inattention, hyperactivity, and impulsivity, beginning in early childhood. Attention deficit hyperactivity disorder has a complex etiology based on neurobiological foundations, involving genetic, environmental, and biological factors in the early development process. The etiology of attention deficit hyperactivity disorder has not been completely clarified yet, but it has been suggested that increased oxidative stress is one of the possible common etiologies in attention deficit hyperactivity disorder. Oxidative stress can cause cellular damage, DNA repair system malfunction, and mitochondrial dysfunction. Mitochondrial dysfunction is thought to be a susceptibility factor in the development of psychiatric diseases. This article aims to review the research conducted to evaluate the possible relationship between attention deficit hyperactivity disorder and mitochondrial dysfunction and systematically examine the data obtained from these studies. Although studies considering the relationship between attention deficit hyperactivity disorder and mitochondrial dysfunction are less than those of autism spectrum disorder, schizophrenia, and mood disorders, studies on attention deficit hyperactivity disorder are increasing. A compensating system against mitochondrial dysfunction caused by hereditary and environmental factors may be generated by an increase in mitochondrial DNA copy number. Mitochondrial DNA copies may decrease with the reduction of attention deficit hyperactivity disorder severity and attention deficit in patients receiving treatment and may positively affect mitochondrial functions. The literature data of this review show that mitochondrial dysfunction could be a crucial factor in the pathophysiology of attention deficit hyperactivity disorder. Understanding mitochondrial contributions in the pathogenesis of attention deficit hyperactivity disorder may result in new diagnostic tools and the development of new therapeutic strategies for attention deficit hyperactivity disorder treatment.

**Main Points**

- Mitochondrial dysfunction may be a possible contributing factor to the etiology of attention deficit hyperactivity disorder (ADHD).
- Studies have shown mitochondrial genetic variations in ADHD cases such as increased mitochondrial DNA (mtDNA) copy number, mtDNA mutation and polymorphisms, and mtDNA haplogroups.
- However, the genetic basis of mitochondrial dysfunction in ADHD patients remains uncertain.
- Extended follow-up and post-mortem studies of brain tissue, cell/tissue type-specific imaging studies, and well-designed animal studies will provide more insight into mitochondrial dysfunction in ADHD.

## Introduction

Attention deficit hyperactivity disorder (ADHD) is a lifelong neurodevelopmental disorder that manifests itself in early childhood as inattention, hyperactivity, and impulsivity.^1^ As high as 5.29% and 5.9%-7.1% of children and adolescents worldwide are diagnosed with ADHD.^2^ It usually persists in adulthood with a frequency of 2.5%.^3^ Attention deficit hyperactivity disorder has a complex etiology based on neurobiological foundations, involving genetic, environmental, and biological factors in the early development process.^4^ The etiology of ADHD has not been completely clarified yet, but it has been suggested that elevated oxidative stress is one of the most widely known etiologies in ADHD.^5^

Normal oxidation–reduction reactions that occur during cell metabolism lead to hazardous compounds named reactive oxygen species (ROS). Organisms produce anti-oxidants to counter the damaging effects of ROS. When the amount of anti-oxidants generated is inadequate to offset the detrimental effects of ROS, oxidative stress arises.^6^ Increased oxidative stress has been linked to cellular damage, DNA repair system malfunction, and mitochondrial dysfunction (MD).^7^ Brain tissue is sensitive to oxidative stress caused by the action of free radicals. The reason for this is that, despite the brain’s high energy demands, its anti-oxidant defense remains low.^8^ Oxidative stress has been linked to a variety of mental illnesses, including schizophrenia, bipolar disorder (BD), major depressive disorder (MDD), and anxiety disorders, according to the literature.^9^ Furthermore, oxidative stress can be exacerbated by genetic and environmental risk factors, leading to the development of ADHD.^5^ Although the research on oxidative stress in ADHD is contradictory, numerous studies have found that this group had higher oxidative stress indices.^10–13^ In a meta-analysis that investigated the relationship between oxidative stress and ADHD, consisting of 231 ADHD patients and 6 studies, oxidative stress in ADHD increased, and some symptoms of ADHD regressed with anti-oxidant treatment.^6^

Mitochondria are sophisticated organelles that contribute to the generation of adenosine triphosphate (ATP) in the electron transport chain through oxidative phosphorylation (ETC).^14^ Mitochondria are also engaged in other biological functions of cells, such as cell cycle control, signaling, differentiation, apoptosis, regulation of membrane potential, and calcium regulation.^15,16^ Mitochondria are highly sensitive to ROS. It has been determined that pyruvate dehydrogenase activity decreases with oxidative stress,^17^ superoxide formed as a result of stress disrupts the activity of complexes I, III, and V, and ATP production decreases as a result of MD.^18^ In addition, it has been proven that complexes I and III located in the mitochondria increase oxidative stress by producing ROS.^17,19^ As a result, oxidative stress can cause MD, but at the same time, MD can increase ROS and cause an increase in oxidative stress.

However, MD is often difficult to measure and demonstrate. The Mitochondrial Medicine Society advised using a biopsy of the affected tissue to determine MD.^20^ Next-generation sequencing of the mitochondrial DNA (mtDNA) genome allows detecting variations in the mtDNA as well as the mutation loads.^21^ The biopsy can also be dyed for histological analysis or utilized to figure out how oxidative phosphorylation occurs. Although tissue biopsy is the standard method, these diagnostic methods are not always accessible and useful, as it is often impossible to take biopsies from affected tissues, especially brain tissue. Since blood cells can be easily obtained from all patients, it will be more convenient and easier to use as a surrogate of biopsy tissue.^22^

**Figure 1.**
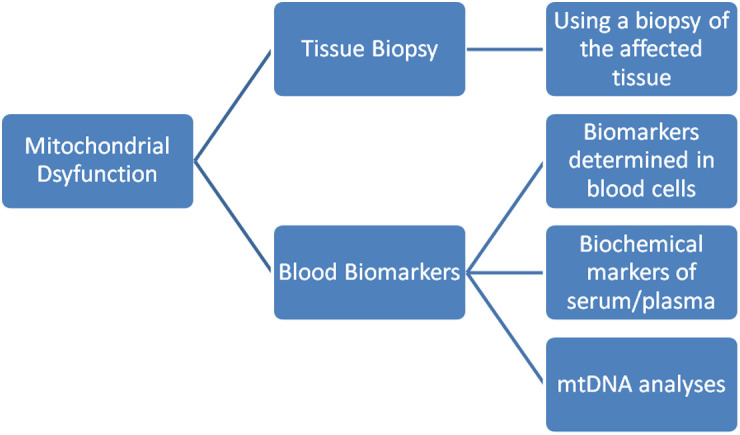
Summary of diagnostic approaches to mitochondrial dysfunction. Adapted from Hubens et al.^22^

Mitochondrial dysfunction’s blood biomarkers can be classified into 3 groups^22^:

1. Biomarkers determined in blood cells: It is a method of measuring the function of oxidative phosphorylation and the mitochondrial membrane potential in blood cells, namely lymphocytes, platelets, and peripheral blood mononuclear cells. These biological biomarkers have the distinct benefit of being able to monitor mitochondrial activity in real-time. The downsides of this approach, on the other hand, are that it is complicated, difficult to standardize, and requires the use of living cells to assess membrane potential.
2. Biochemical markers of serum/plasma: This is a technique for determining energy-related metabolites such as lactate, pyruvate, creatine, amino acids, and the fibroblast growth factor-21 and growth differentiation factor 15. Testing biomarkers in the bloodstream is technically simpler and more accessible than measuring biomarkers in blood cells. To detect mitochondrial diseases, certain biomarkers are frequently utilized. However, this approach has the drawback of measuring MD indirectly and being difficult to standardize.
3. Mitochondrial DNA analyses: Because it may not be possible to isolate and culture viable cells from every patient, mtDNA analysis may be an appealing alternative to cellular analysis. With popular procedures like real-time polymerase chain reaction and sequencing, extracted mtDNA may be preserved for a long time and evaluated as needed. Because just a little quantity of DNA genome is required for analysis, only a few clinical samples are required.^23^ This approach has the disadvantage of not being as precise as functional measurements. Figure 1 depicts methods for identifying MD.

Despite studies stressing the possible role of ROS in the development of ADHD, the concept of oxidative stress’s activity in the pathogenesis of ADHD at the cellular level remains unknown. The most plausible pathway for oxidative stress to contribute to the pathophysiology of ADHD has been proposed as MD.^24^ A number of explanations have been proposed to explain the link between MD and ADHD. The first is that increasing oxidative stress damages mtDNA, resulting in MD. By degrading lipids, proteins, and nucleic acids and affecting membrane lipid integrity, prolonged oxidative stress alters protein and chromatin structures, resulting in MD, apoptosis, and eventually ADHD.^25,26^ The second is oxidative stress, which happens during the synthesis of mitochondrial energy. In the mitochondria, neuronal structures require a lot of energy and are vulnerable to oxidative stress. However, ROS such as superoxide anions and hydrogen peroxide (H_2_O_2_) cannot be effectively removed because of the MD that happens during this high generation. High levels of ROS can cause astrocytes and microglia to become activated. The release of proinflammatory chemokines and cytokines rises as a result of this activation, creating a vicious cycle.^27^ This vicious circle may increase the risk of ADHD pathogenesis.^28^ Third, increased H_2_O_2_ due to MD may play a functional role in ADHD by suppressing dopamine release in the frontostriatal pathway.^29^ This pathway may be one of the possible mechanisms underlying dopamine deficiency in the frontostriatal pathway in ADHD. The fourth is that lipid peroxidation pathology resulting from MD disrupts neuronal cell membranes. Impairment of the permeability of dopaminergic and serotonergic neurotransmitter systems due to damaged membranes may prevent reaching the required amount of neurotransmitters and affect ADHD development.^30^

Mitochondrial dysfunction has been implicated in the pathogenesis of a variety of mental diseases.^31-33^ Several studies have linked MD to schizophrenia,^34,35^ BD,^36,37^ MDD,^38,39^ and autism spectrum disorder (ASD).^40^ However, there is a scarcity of data on the link between MD and ADHD.^41^

The main purpose of this narrative review is to present an overview of ADHD and MD investigations in order to shed light on the pathophysiology of ADHD, as MD may play a part in it.

## Materials and Methods

**Figure 2.**
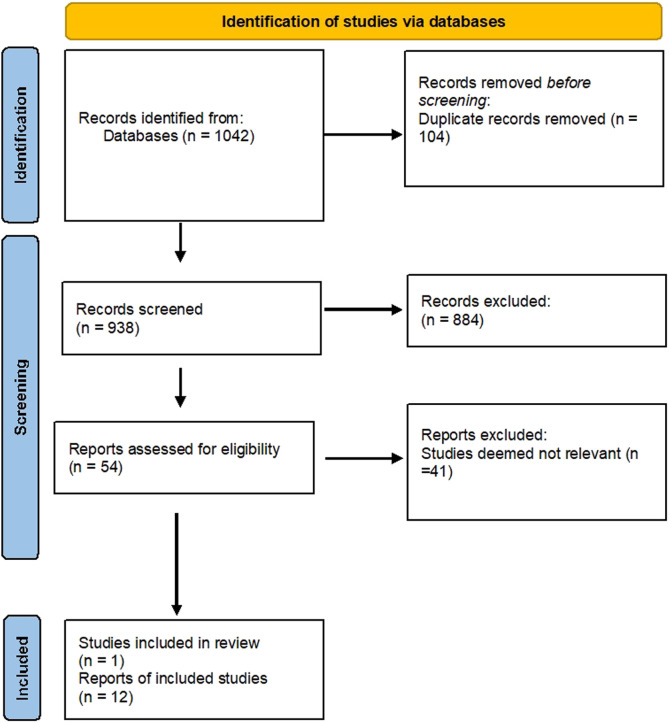
Flow diagram of the selected articles.

Search terms relating to “Attention Deficit Hyperactivity Disorder,” “ADHD,” “mitochondrial dysfunction,” “Mitochondrial DNA,” and “mtDNA” were combined. Articles included in this study were searched in the PubMed/Medline, Google Scholar, and Scopus databases with combined search terms up to August 2022. Peer-reviewed studies conducted exclusively on ADHD and MD and articles published in English were included in the review. There were no restrictions on the year of publication or the methodologies used in order to conduct a complete evaluation of the literature. The flow diagram summarizing the literature review is shown in Figure 2. The following domains were used to structure the discussion of the papers: mitochondria dysfunction in people with ADHD, mtDNA copy number in ADHD, cybrid cell lines, nuclear mitochondrial genetic variation, and induced pluripotent stem cells (iPSC)-based studies of MD in ADHD, mtDNA mutation and polymorphism in ADHD, association between mtDNA haplogroup and risk of ADHD, mitochondrial-associated protein biomarkers in ADHD, and the cell danger response.

### Mitochondria Dysfunction in People with Attention Deficit Hyperactivity Disorder

As stated earlier, MD may play an essential role in causing or increasing susceptibility to ADHD. This section reports the recent scientific papers supporting this hypothesis and summarizes the evidence. The findings of all studies on this subject are summarized in Table 1.

### Cybrid Cell Lines, Nuclear Mitochondrial Genetic Variation, and Induced Pluripotent Stem Cells-Based Studies of Mitochondrial Dysfunction in Attention Deficit Hyperactivity Disorder

Verma et al^41^ evaluated the activities of cytoplasmic hybrid neuroblastoma cells (also known as cybrid cells) created by combining platelets from ADHD patients and healthy controls with neuroblastoma cells lacking mitochondria in research. Decreased cellular and mitochondrial respiration, lower ATPase 6/8 transcript levels, reduced mitochondrial complex V activity, loss of mitochondrial membrane potential, and increased oxidative stress were seen in ADHD hybrids compared to healthy controls in this study. The research is the first to suggest that MD may have a role in the pathophysiology of ADHD as an inherited component.

Most genes that regulate mitochondrial functioning are encoded by nuclear DNA (nDNA).^42^ The systematic review by Giannoulis et al^43^ included a wide variety of studies, ranging from large sample genome-wide association studies to human-induced pluripotent stem cells (hiPSCs) and rat models, examining the relationship of nuclear mitochondrial genes with ADHD. By adding genes or proteins, hiPSCs are created from somatic cells by artificial reprogramming.

The first of these studies was conducted by Lesch et al^44^ to address micro-deletion and macro-duplications in DNA in 99 children with ADHD. In this study, deletions were found in nicotinamide adenine dinucleotide (NADH) dehydrogenase one alpha subcomplex assembly factor and uncoupling protein 2 mitochondrial genes in ADHD cases. In addition, 924 ADHD–parent–sibling triads were recruited in 2014 by Lee and Song^45^ to identify both the genetic pathway that contributes to the ADHD phenotype and the single-nucleotide polymorphisms. In this study, the mitochondrial apoptotic signaling pathway was the second most substantial pathway in ADHD cases.

Indirect evidence from pharmacological studies using methylphenidate in animal has demonstrated that the drug affects the activity of mitochondrial ETC enzymes, thereby correcting the energy deficiency in the brain.^46^ In addition, in another animal study to determine the effects of long stimulants used in the chronic treatment of ADHD, it was shown that mitochondrial organization, apoptosis, and cell adhesion genes were altered in the striatum and prefrontal cortex of ADHD (spontaneously hypertensive rats) rat models compared to the control group.^47^

In addition to animal studies to identify genetic changes underlying the development of ADHD, Palladino et al^48^ used human-derived fibroblasts and hiPSCs-generated dopaminergic neurons from ADHD patients to examine PARK2, a potential gene for ADHD that encodes a protein involved in mitochondrial quality regulation. The parkin protein is encoded by the PARK2 gene (also known as PRKN). They suggest that ADHD PARK2 copy number variation (CNV) carriers might have an energy impairment. This disruption could be due to the different roles of PARK2 in mitochondrial functioning, potentially leading to the impairment of normal brain plasticity and cellular resilience.^49^ During the sensitive developmental stage, MD may play a role in the development of ADHD by negatively affecting nervous system maturation. In addition, they indicate that chemicals that impact mitochondrial activity (anti-oxidants) might be used as therapy options based on their findings.^48^

### Mitochondrial DNA Copy Number in Attention Deficit Hyperactivity Disorder

Mitochondria carry their genome (called mtDNA), which is thought to be a possible biomarker for MD, and mtDNA is very susceptible to oxidative stress’s negative effects.^50^ Mitochondrial DNA is a 16.569 bp circular double-stranded molecule with 37 genes that code for critical ETC enzyme complex proteins and partial components of the intramitochondrial protein synthesis mechanism. Due to a lack of protective histones and low DNA repair ability, mtDNA is more vulnerable to intramitochondrial ROS than nDNA.^51^ The regulation of mtDNA copy number is critical for meeting the energy demands of high-energy cells, such as neurons. As a result, an increase in mtDNA copy number may be produced as a defense mechanism against MD caused by genetic and environmental causes.^52^

Many studies have found that the mtDNA copy number in lymphocytes in both brain and peripheral tissue rises, leading to an increase in MD and oxidative stress.^50,53^ When compared to the study of organs with high energy demand, such as liver or muscle, mtDNA copy number analysis in the blood of individuals with primary MD has shown poorer sensitivity and specificity.^54^ For example, Dimmock et al^55^ found that mtDNA copy counts in muscle tissue had a specificity of 89%, whereas blood had a specificity of 74%. Despite this limitation, changes in mtDNA copy number in multifactorial disorders may still signal probable mitochondrial abnormalities.^22^ Because PMBCs may vary their expression patterns in response to physiological and environmental stimuli and reach the whole body and organs, they can be utilized as a surrogate model for changes in tissues like muscle and brain cells.^56^

Kim et al^57^ compared mtDNA copy number and methylation ratio of the D-loop region, as well as the peroxisome-proliferator-activated receptor co-activator-1α (PPARGC1A) between ADHD patients and controls, as well as among ADHD presentations, in a study with a total of 140 participants aged 6-16 years, 70 with ADHD and 70 in the control group. The study looked at mtDNA copy number, methylation of the D-loop region, and PPARGC1A, which are all considered biomarkers for MD. While the ADHD group had higher mtDNA copy numbers and lower mtDNA methylation ratio of PPARGC1A than the healthy controls, there was no change in the methylation ratio of the D-loop region. After controlling the intelligence quotient level, the only difference between the ADHD and control groups was the mtDNA copy number. These findings show that MD may play a role in the pathogenesis of ADHD and that mtDNA is the best biomarker for MD. Furthermore, there were no significant relationships between relative mtDNA copy number and factors on the ADHD Rating Scale or ADHD presentations. This finding revealed that the number of copies of mtDNA had no bearing on the clinical features of ADHD.

Öğütlü et al^58^ discovered that the mtDNA copy number of 56 children with ADHD was considerably greater than that of 56 healthy children in his research. The number of copies of mtDNA in children with ADHD was 1.3 times greater than in healthy children in this investigation, as observed in children with ASD.^59^ The increased mtDNA copy number in ADHD patients, similar to autism reports, may imply that MD plays a role in the pathophysiology of ADHD, according to this study. However, no significant differences in ADHD presentations and mtDNA copy numbers were detected in this investigation, as in Kim et al.^57^

Öğütlü et al^60^ re-examined the severity of ADHD and the effects of treatment on mtDNA in 28 children who were diagnosed with ADHD in his initial research and whose mtDNA copy counts were considerably greater than those of healthy children. This is the first long-term research to look at variations in mtDNA copy number in ADHD patients. The individuals were separated into 2 groups: those who got ADHD treatment and those who did not, and their mtDNA copy number was measured. The average of all patients’ first and second mtDNA copy counts did not vary regardless of treatment, according to the research. However, it has been shown that ADHD index and inattention subscale scores also decreased in the drug-treated group in those with decreased mtDNA, and this relationship was significant for the ADHD index. This finding suggests that mtDNA copies may decrease with the reduction of ADHD severity and attention deficit in patients receiving treatment and may positively affect mitochondrial functions. The findings of this study suggest that MD may have a function in the pathophysiology of ADHD that is independent of treatment.

### Mitochondrial DNA Mutation and Polymorphism in Attention Deficit Hyperactivity Disorder

Initial evidence of sporadic mtDNA mutations in ADHD is mostly based on case reports.^61-63^ These case reports include monogenetic diseases that exhibit the ADHD phenotype or mimic ADHD rather than primary ADHD. However, they are precious because they are the first case reports showing mtDNA mutation in ADHD.

Mitochondrial dysfunction triggered by mtDNA polymorphisms has been linked to a variety of disorders, including type 2 diabetes, breast, and esophageal cancer.^64,65^ Within NADH dehydrogenase subunit 3 complex I, which is involved in mtDNA ETC, the mtDNA 10398 A/G polymorphism causes a non-conservative amino acid shift from threonine (A allele) to alanine (G allele). The 10398 A/G polymorphism of mtDNA influences the pH and calcium levels in the mitochondrial matrix, which are important for glycolytic energy generation. Furthermore, it has been linked to mental disorders such as schizophrenia and BD.^66^

Hwang et al^67^ published the first investigation on the genetic link between mtDNA polymorphisms and ADHD in Korea in 2017. This study found a strong link between the mtDNA 10398 A/G polymorphism and ADHD. They also discovered that the mtDNA 10398 A/G polymorphism was linked to aggressiveness and leadership behavior scores in ADHD males. These findings imply that the mtDNA 10398 A/G polymorphism may have a role in the development of ADHD.

### Association Between Mitochondrial DNA Haplogroup and Risk of Attention Deficit Hyperactivity Disorder

The human population is categorized into different mtDNA haplogroups (namely, a group of mtDNAs that share specific ancient variants) based on cumulative mtDNA variants during the origin and immigration of modern human beings.^68^ Prior studies indicate that mtDNA haplogroups show various metabolic capacities and play an essential role in multiple diseases and features like cancers, neurodegenerative diseases, and longevity.^69,70^ In addition, there are studies that mtDNA haplogroups contribute to the development of psychiatric disorders in different populations.^71,72^

The study conducted by Hwang et al^73^ to research the role of mtDNA haplogroups on ADHD and the effect of mtDNA haplogroups on the development of ADHD subtypes in Korean children included 472 participants. This is the first study to address the relationship between ADHD and mtDNA haplogroups. In this study, boys and girls were evaluated separately, considering that ADHD is more common in boys than girls. As a result of the research, it was determined that mtDNA haplogroup B4 was associated with both genders, and mtDNA haplogroup D4b was associated only with girls in developing ADHD symptoms. In addition, mtDNA haplogroup B5 was a protective factor for ADHD in Korean children. In this study, subtype analysis demonstrated that haplogroup B4 was strongly associated with the ADHD combined subtype, and haplogroup F was related to the ADHD inattentive subtype. These results suggest that mtDNA haplogroups may have a role in Korean children’s genetic etiology of ADHD symptoms.

Chang et al^74^ studied ADHD patients and controls in European populations to determine whether mtDNA haplogroups affect ADHD risk. This is the first large-scale study to research the role of mtDNA haplogroups in ADHD in European people. As a result of the analysis of 3 independent European cohorts, it was found that mtDNA haplogroups K and U were associated with a reduced risk of ADHD, whereas super haplogroup HHV* was associated with an increased risk of ADHD. The results obtained from the study supported those genetic variations of mtDNA play a role in the development of ADHD.

### Mitochondrial-Associated Protein Biomarkers in Attention Deficit Hyperactivity Disorder

Mitochondrial proteins are important regulators of the brain’s bioenergetic process and apoptosis, and their alterations can contribute to neurodevelopmental disorders.^75^ HtrA2, Park7, and α-synuclein are 3 mitochondrial proteins implicated in the pathophysiology of Parkinson’s disease (PD)^76^ and take an active part in the developing brain via many possible pathways.^76^ Attention deficit hyperactivity disorder and PD may share neurobiological processes, such as dopamine system impairment, which may play a role in both disorders’ pathogenesis.^77^

Lee et al^75^ conducted the first study in 2019 with ADHD patients to investigate the link between mitochondrial proteins (HtrA2, α-synuclein, and Park7) and ADHD. Girls with ADHD had greater plasma HtrA2 levels than girls without ADHD, and their HtrA2 levels were favorably associated with comprehensive verbal ability and negatively associated with behavioral symptoms, according to this study. However, they found no associations between these mitochondrial proteins, cognitive results, or clinical symptoms in boys. There were also no changes between ADHD patients and healthy controls for α-synuclein and Park7. These findings suggest that the pathophysiology of ADHD may be influenced by an underlying gender-specific mitochondrial mechanism.

### The Cell Danger Response

Cell danger response (CDR) is an ancient and global response to danger, stress, or injury.^78^ Cell danger response refers to whole responses to stress, inflammation, immunology, metabolism, microbiota, epigenetics, behavior, and memory. Mitochondria are especially suited to observing environmental and genetic conditions, as well as gene–environment (echogenic) interactions, which regulate CDR. Mitochondria control the CDR by monitoring and reacting to physical, chemical, and microbiological conditions within and outside the cell. As a result, mitochondria connect cellular and environmental health. Although the CDR is a biological reaction, it has the potential to alter the human mind and behavior, child development, physical fitness and resilience, fertility, and population vulnerability to disorder.^79^ It is stated that many neurodevelopmental, autoimmune, and degenerative disorders may occur due to failure to regulate the CDR.^79^ Although a study evaluating the relationship between CDR and autism has been done recently^80^ and promising results have been obtained, there is no study with ADHD on this subject yet. Understanding CDR regions, a metabolic response that protects cells from harm is critical to understanding the pathophysiology of neurodevelopmental disorders including ASD and ADHD.

## Conclusion

The purpose of this review is to summarize recent studies evaluating the impact of MD on the pathophysiological mechanisms of ADHD patients. Although studies considering the relationship between ADHD and MD are less than those of ASD, schizophrenia, and mood disorders, studies on ADHD are increasing. The literature data of this review show that MD may be a key mechanism underlying the pathophysiology of ADHD. However, the genetic basis of MDs in ADHD patients remains uncertain. Extended follow-up and postmortem brain tissue studies with whole-exome sequencing tests in larger samples regarding mtDNA in ADHD patients are essential in elucidating the effect of MD on ADHD and the genetic mechanism in the etiology of ADHD. Large-scale genetic studies, including common variants and rare CNVs, as well as iPSC-based studies, will also help to evaluate the role of metabolic gene regulation in ADHD. In addition to genetic studies, cell/tissue type-specific imaging studies are needed to evaluate metabolic function in ADHD patients. In addition, it is a matter of debate how much the findings obtained from peripheral tissues can represent ADHD, a brain disease. Especially with well-designed animal studies on this subject, important data can be determined by measuring mitochondrial changes in peripheral and brain tissues by creating ADHD-like symptoms in animal models. Further studies with larger cohorts and long-term follow-up researching the relationship between clinical features and biomarkers of MD are expected to help clarify the clinical role of MD in ADHD and determine what changes in MDs occur at different stages of ADHD. Similarly, studies in animal models can provide information about the effects of stimulants, atomoxetine, and anti-oxidant drugs on MD in ADHD.

## Figures and Tables

**Table 1.** Main Findings of Studies Assessing the Mitochondrial Dysfunction in Patients with ADHD

References	Samples	Age	Sex	Race/Ethnicity	Type of Biospecimens	Methods	Results
Verma et al (2016)^41^	3 ADHD, 4 control	5-13 years	N/A	N/A	Human platelet	Cybrid cell lines generated from human platelet—neuroblastoma cell line	ADHD group showed significantly lower cellular and mitochondrial respiration, reduced ATPase transcript levels, reduced mitochondrial complex activity, loss of mitochondrial membrane potential, and elevated oxidative stress than controls
Lesch et al (2011)^44^	99 ADHD	N/A	78 male/21 female	Caucasian	PMBCs	Genome-wide copy number analysis in ADHD cases with high-resolution (aCGH)	Mitochondrial gene NDUFAF2 and UCP2 deletion were observed in ADHD cases
Lee and Song (2014)^45^	924 ADHD–parent–sibling trios, 2758 total individuals	Children and siblings aged 6-17 years, N/A for parents	N/A	Caucasian	PMBCs	GWAS pathway analysis in ADHD–parent–child trios	Mitochondrial apoptotic signaling gene pathway was identified as the second strongest mechanism in ADHD cases
dela Penã et al (2014)^47^	ADHD SHR rats and controls	N/A	N/A	N/A	Brain tissue	Gene alterations in the PFC and striatum were determined by qRT-PCR.	Mitochondrion organization genes were downregulated in the striatum of ADHD rat models treated with methylphenidate
Palladino et al (2020)^48^	4 ADHD (3 CNV carriers+ 1 wild type), 2 control	28-47	1 male/5 female	Caucasian	HDF + hiPSC	Midbrain dopaminergic neurons generated from fibroblast-derived iPSCs (with a CNV in the PARK2 locus)	PARK2 CNV carriers with ADHD have an energy impairment, which might impact neuronal development
Kim et al (2019)^57^	70 ADHD, 70 control	6-17 years	88 male/52 female	Asian	PMBCs	mtDNA analysis was performed with qPCR, and the methylation ratio was measured using MSP after bisulfite conversion	Only the mtDNA copy number was significantly higher in ADHD than controls after adjusting for IQ level
Öğütlü et al (2020)^58^	56 ADHD, 56 control	6-16 years	72 male/40 female	Caucasian	PMBCs	mtDNA analysis was performed with qPCR	mtDNA copy number was significantly higher in ADHD than controls
Öğütlü et al (2021)^60^	28 ADHD	6-16 years	15 male/13 female	Caucasian	PMBCs	mtDNA analysis was performed with qPCR	mtDNA copy number did not change in patients with ADHD over a period of 1 year regardless of treatment
Hwang et al (2017)^67^	150 ADHD, 322 control	7-10 years	288 male/184	Asian	PMBCs	mtDNA 10398 A/G polymorphism was genotyped using a PCR-RFLP	mtDNA 10398 A/G polymorphism was significantly associated with the ADHD children
Hwang et al (2019)^73^	150 ADHD, 322 control	7-10 years	288 male/184	Asian	PMBCs	mtDNA haplogroups determination was performed with 20-plex SNaPshot assay	mtDNA haplogroup B4 is associated with ADHD in both sexes, while mtDNA haplogroup D4b is associated with ADHD in girls only
Chang et al (2020)^74^	2076 ADHD, 5078 control	6-18 years	N/A	Caucasian	PMBCs	mtDNA haplogroup was determined by HaploGrep 2	mtDNA haplogroups K and U are associated with a reduced risk of ADHD, while super haplogroup HHV* is associated with an increased risk of ADHD
Lee et al (2019)^75^	125 ADHD, 66 control	6-16 years	141 male/50 female	Asian	PMBCs	HtrA2, α-synuclein, and Park7 plasma levels were determined using Luminex assay	The girls with ADHD demonstrated higher plasma HtrA2 levels than control girls

aCGH, array comparative genomic hybridization; ADHD, attention deficit hyperactivity disorder; CNV, copy number variation; GWAS, genome-wide association studies; HDF, human-derived fibroblast; hiPSC, human-induced pluripotent cells; IQ, intelligence quotient; MSP, methylation-specific PCR; mtDNA, mitochondrial DNA; N/A, not applicable; NDUFAF2, nicotinamide adenine dinucleotide dehydrogenase 1 alpha subcomplex assembly factor; PCR-RFLP, reaction-restriction fragment length polymorphism; PFC, prefrontal cortex; PMBCs, peripheral blood mononuclear cells; qPCR, quantitative PCR; qRT-PCR, quantitative real time PCR; SHR, spontaneously hypertensive rats; UCP2, uncoupling protein 2.
